# Impact of germline predisposition genes to hematologic malignancies on transplant outcomes and donor selection

**DOI:** 10.3389/fimmu.2026.1880348

**Published:** 2026-07-28

**Authors:** Zhihui Li, Keyan Yang, Caiyan Zhang, Xianxuan Wang, Lei Wang, Jing Li, Xiaopei Wen, Qian Fei, Jiahong Zhai, Yan Zhou, Zongyang Ming, Yanzhi Song, Yongqiang Zhao, Tong Wu, Qinlong Zheng

**Affiliations:** 1 Department of Bone Marrow Transplantation, Beijing Gobroad Boren Hospital, Beijing, China; 2 Department of Molecular Diagnostic Laboratory, Beijing Gobroad Boren Hospital, Beijing, China

**Keywords:** donor selection, germline mutations, hematopoietic stem cell transplantation, inborn errors of immunity, prognosis

## Abstract

**Introduction:**

Germline predisposition gene mutations may influence outcomes in patients undergoing hematopoietic stem cell transplantation (HSCT).

**Methods:**

This retrospective study of 558 patients undergoing HSCT (allo-HSCT in 557 cases, auto-HSCT in 1 case) evaluated the impact of germline predisposition gene mutations on outcomes.

**Results:**

Enrichment of *HAVCR2* mutations in B-NHL and *UNC13D* mutations in MDS was associated with poorer prognosis. While homozygous mutation carriers (7.5%) overall did not have reduced survival, their outcomes were significantly better with unrelated versus related donors (3-year DFS/OS: 82.1% vs. 48.3%/56.7%). Mutations in genes linked to inborn errors of immunity affecting both cellular and humoral immunity (IACHI) predicted inferior survival.

**Conclusion:**

Germline mutations, particularly in IACHI-related genes, influence HSCT outcomes. Pre-transplant genetic screening and opting for unrelated donors for homozygous carriers may optimize transplantation strategies.

## Introduction

There is increasing recognition that the development of hematologic malignancies is closely associated with genetic predisposition (1, 2). Germline mutations in genes involved in immunity, DNA repair, and tumor suppression have been linked to the initiation and progression of both pediatric and adult hematologic malignancies (2–4). Allogeneic hematopoietic stem cell transplantation (HSCT) remains a potentially curative option for many relapsed or refractory hematologic diseases (5); however, its efficacy varies substantially depending on disease-related, patient-related, and donor-related factors (6).

With the increasing integration of genomic sequencing into clinical oncology and hematology, numerous germline genetic variants, also referred to as cancer predisposition gene variants, have been identified in patients with cancer and their family members (1). In hematologic malignancies, familial aggregation was first reported in several families with chronic lymphocytic leukemia (CLL) and acute myeloid leukemia (AML) (7).

Therefore, since 2016, the World Health Organization (WHO) classification of hematopoietic neoplasms has included a distinct category termed myeloid neoplasms with germline predisposition (2, 8). However, recent studies have demonstrated that genetic predisposition to hematologic malignancies is not limited to patients with myeloid lineage origins, but also involves lymphomas and plasma cell–related neoplasms (9, 10). Germline mutations in certain genes have been reported to increase the risk of all types of malignancies (e.g., *TP53*, *ATM*, *CDKN2A*, and *CHEK2*), whereas mutations in some germline predisposition genes are more closely associated with hematologic malignancies (e.g., *GATA2*, *RUNX1*, *DDX41*, *ETV6*, *CEBPA*, *ANKRD26*, *SAMD9*, and *SAMD9L*) (11). Identification of germline genetic predisposition has important clinical implications for patients undergoing HSCT, including donor selection and/or conditioning regimen selection for HSCT, as well as guidance for appropriate treatment, and for their family members with respect to screening and genetic counseling.

However, systematic evaluation of these germline predisposition gene variants in HSCT cohorts with hematologic malignancies remains limited, and their impact on donor selection has not been fully investigated. The present study aimed to analyze the profiling of germline predisposition mutations in pediatric and adult patients undergoing HSCT at our center and to evaluate their impact on clinical outcomes.

## Methods

### Patients

Between December 2017 and February 2024, a total of 558 patients underwent HSCT at Beijing Gobroad Boren Hospital (allo-HSCT in 557 cases, auto-HSCT in 1 case). The median age was 16.14 years. The diagnoses included 225 cases of acute B lymphoblastic leukemia (B-ALL), 8 cases of acute B lymphoblastic leukemia/lymphoma (B-ALL/LBL), 37 cases of acute T lymphoblastic leukemia (T-ALL), 33 cases of acute T lymphoblastic leukemia/lymphoma (T-ALL/LBL), 157 cases of AML, 18 cases of myelodysplastic syndrome (MDS), 22 cases of B-cell non-Hodgkin lymphoma (B-NHL), 14 cases of aplastic anemia (AA), 10 cases of acute leukemia (AL), 8 cases of NK/T-cell lymphoma (NKTCL), 4 cases of anaplastic large cell lymphoma (ALCL), 4 cases of chronic active Epstein–Barr virus infection (CAEBV), 4 cases of T-cell non-Hodgkin lymphoma (T-NHL), 3 cases of hemophagocytic lymphohistiocytosis (HLH), 2 cases of chronic myeloid leukemia (CML), 2 case of multiple myeloma, 2 case of chronic NK cell lymphoproliferative disorder, 1 case of blastic plasmacytoid dendritic cell neoplasm, 1 case of globoid cell leukodystrophy, 1 case of myeloid sarcoma, 1 case of Juvenile Myelomonocytic leukemia, 1 case of granulocytic sarcoma, and 1 case of chronic myelomonocytic leukemia. Written informed consent was obtained from all participants or their legal guardians. This study was conducted in accordance with the Declaration of Helsinki and was approved by the Medical Ethics Committee of Beijing Gobroad Boren Hospital (Approval number: KY2020-042-001). All 558 patients underwent germline predisposition gene screening prior to transplantation.

### Conditioning Regimens

All patients received myeloablative conditioning (MAC) regimens prior to hematopoietic stem cell transplantation. A total of 290 patients (52.0%) received a total body irradiation (TBI)-based regimen, consisting of fractionated TBI (total dose 1000 cGy for first transplantation or 800 cGy for second transplantation) combined with other agents. The remaining 267 patients (47.8%) received a busulfan (BU)-based regimen, which included intravenous BU (0.8 mg/kg every 6 hours for 3 days for adults, with pediatric doses adjusted by body weight) in combination with other agents. Semustine (250 mg/m²) was administered on day –3 in all patients. For patients receiving haploidentical or unrelated donor transplants, rabbit anti−human T−cell immunoglobulin (ATG−F, Astellas Pharma, Japan) was added at a total dose of 20 mg/kg, given in divided doses from day –5 to day –2.

### GVHD prophylaxis and supportive care

For GVHD prophylaxis, all patients received a combination of cyclosporine, mycophenolate mofetil, and short−course methotrexate. Cyclosporine was administered intravenously at 1.25 mg/kg every 12 hours starting from day –9 and was switched to oral administration after one month. Methotrexate was given intravenously at 15 mg/m^2^ on day +1 and 10 mg/m^2^ on days +3, +6, and +11. Acute GVHD was graded according to the modified Glucksberg criteria as reported by Przepiorka et al. (12, 13), with grade III–IV considered severe. Chronic GVHD was assessed using the NIH consensus criteria (14). Antifungal prophylaxis (echinocandin or triazole agents) was given as primary or secondary prophylaxis based on each patient’s history. Antiviral prophylaxis included acyclovir for herpesvirus infections and trimethoprim−sulfamethoxazole for *Pneumocystis jirovecii* pneumonia (PCP). Viral monitoring was performed by quantitative PCR for human herpesviruses 1–8, BK virus, and JC virus; plasma CMV and EBV DNA levels were monitored weekly or twice weekly post−transplant. Viremia was defined as detectable virus in blood, and viral disease was defined as virus infection in tissues or organs with clinical symptoms or organ damage (e.g., viral cystitis caused by BK virus, JC virus, or adenovirus after HSCT).

### Detection of germline predisposition gene mutations

Before transplantation, the peripheral blood mononuclear cells (PBMCs) of patients were collected to detect predisposition gene mutations related to hematologic malignancies or immune system diseases by targeted sequencing (900+ genes). Genomes were extracted from PBMCs before HSCT using the TIANamp blood DNA kit (Tiangen Biotechnology Co.). The extraction was performed according to the manufacturer’s instructions. The extracted DNA concentration was determined using a Nanodrop 2000 (Thermo Fisher). The genomic DNA library was constructed using KAPA HyperPlus Kits (Roche). Library concentration was measured using the Qubit 4.0 Fluorometer (Thermo Fisher). The constructed DNA library was captured using the NimbleGen SeqCap EZ Library SR (Roche) kit, and captured using an Agilent SureSelect XT Human All Exon v5. The captured library was sequenced using the NextSeq550 (Illumina) sequencer, and the sequencing was performed using the PE150 program according to the manufacturer’s instructions. For WES average sequencing depth after deduplication was 100×. All the germline variants (VAF >15%, MAF <5%, predicted moderate or high impact) were subsequently validated by Sanger sequencing using genomic DNA extracted from non−hematopoietic tissues (oral epithelial cells or nail clippings) from the same patient. Only variants confirmed as germline by this method were included in the final analysis.

### Bioinformatics analysis

Germline variant calling was performed with the Broad Institute’s Genome Analysis Toolkit (GATK). The variant list was further filtered to select variants of approximately 700 genes that were associated with hematological tumors and immunodeficiency. Germline variants with an allele frequency >15% in our samples and with a predicted moderate or high impact on protein function were further filtered to select variants according to their frequency in the genomAD, ExAC and 1000G databases (minor allele frequency (MAF) < 5%). Integrative Genomics Viewer (IGV) software was used to manually inspect sequencing results. The candidate genetic risk variants were validated in family members of patients by Sanger sequencing. And the interpretation of variants was based on the HGMD and ACMG guideline (15, 16). We only included pathogenic and likely pathogenic variants for further analysis. Germline inborn errors of immunity (IEI) gene mutations were classified according to the 2022 classification of Human Inborn Errors of Immunity (17).

### Statistical analysis

Statistical analyses were conducted using R version 3.6.2. Data are expressed as the median and interquartile range (IQR). The Wilcoxon rank-sum test was applied to assess differences between two groups; the Kruskal-Wallis test was used to analyze differences among more than two groups. Pearson’s chi-squared test or Fisher’s exact test was employed to compare differences between categorical variables. Gray’s test or Log-rank test, and Kaplan-Meier curves were used to compare relapse and OS. All survival analyses were performed using the “survminer” and “survival” packages in R. *P* < 0.05 was considered significant for two-sided tests.

## Result

### Cohort characteristics

This retrospective cohort included 558 patients with hematologic diseases who underwent HSCT at Beijing Gobroad Boren Hospital between December 2017 and February 2024. Peripheral blood samples were collected from all patients before HSCT for germline predisposition gene testing. The last follow-up date was June 30, 2024. The median follow-up duration was 33.8 months (95% CI: 31.6–36.5 months). The mean age of the patients was 16.14 years (range: 0.33–71.43 years), including 298 pediatric patients (<18 years) and 260 adult patients (≥18 years). Male patients accounted for 59.0% of the cohort (n = 329), and female patients accounted for 41.0% (n = 229). The major disease types were B-ALL (40.3%, n = 225), AML (28.1%, n = 157), T-ALL (6.6%, n = 37), T-LBL/ALL (5.9%, n = 33), B-NHL (3.9%, n = 22), MDS (3.2%, n = 18), and AA (2.5%, n = 14). Among the 558 patients, 136 (24.4%) had undergone a previous HSCT. Regarding pre−transplant remission status, 94 patients (16.8%) were in first complete remission (1CR), 224 (40.1%) were in more than first CR (>1CR), and 240 (43.0%) were not in CR. The indications for the 136 second transplants were disease relapse after the first transplant (n=128, 94.1%), graft rejection (n=7, 5.1%), and poor graft function (n=1, 0.7%). Regarding conditioning regimens, 290 patients (52.0%) received a total body irradiation (TBI)–based regimen, whereas 267 patients (47.8%) received a busulfan (BU)–based regimen. Donor types included haploidentical donors in 391 patients (70.1%), matched sibling donors in 41 patients (7.3%), unrelated donors in 125 patients (22.4%), and autologous transplantation in 1 patient (0.2%). Other clinical characteristics of the cohort are summarized in **Table 1**.

**Table 1:** Baseline clinical and transplant characteristics of the cohort (N = 558).

Factor	Level	Overall
		N=558
Sex (%)	Female	229 (41.0%)
	Male	329 (59.0%)
Age (median [IQR])		16.14 [6.50,32.57]
Diagnosis (%)	B-ALL	225 (40.3%)
	AML	157 (28.1%)
	T-ALL	37 (6.6%)
	T-LBL/ALL	33 (5.9%)
	NHL-B	22 (3.9%)
	MDS	18 (3.2%)
	AA	14 (2.5%)
	AL	10 (1.8%)
	B-LBL/ALL	8 (1.4%)
	NKTCL	8 (1.4%)
	ALCL	4 (0.7%)
	CAEBV	4 (0.7%)
	NHL-T	4 (0.7%)
	HLH	3 (0.5%)
	CML	2 (0.4%)
	Others	9 (1.6%)
CNSL (%)	Yes	81 (14.5%)
	No	477 (85.5%)
Relapse before HSCT (%)	Yes	334 (59.9%)
	No	224 (40.1%)
Status before HSCT (%)	1CR	94 (16.8%)
	>1CR	224 (40.1%)
	Not CR	240 (43.0%)
Second HSCT (%)	Yes	136 (24.4%)
	No	422 (75.6%)
HCT-CI (%)	0-1	410 (73.5%)
	2	88 (15.8%)
	3	60 (10.8%)
Conditioning regimen (%)	TBI-based	290 (52.0%)
	BU-based	267 (47.8%)
	others	1 (0.2%)
Donor type (%)	Haplo identical related	391 (70.1%)
	Matched related	41 (7.3%)
	Unrelated	125 (22.4%)
	Auto	1 (0.2%)
Blood match (%)	Yes	279 (50.0%)
	No	274 (49.1%)
	Unknown	5 (0.9%)

The overall 3-year OS of the cohort was 66.3% (95% CI: 62.3–70.6%), the 3-year DFS was 59.2% (95% CI: 55.2–63.6%), the 3-year cumulative incidence of relapse (CIR) was 29.7% (95% CI: 24.2–31.7%), and the 3-year non-relapse mortality (NRM) was 11.1% (95% CI: 7.46–12.4%). The overall incidence of acute GVHD (44.09%, with 31.00% grade I–II and 13.08% grade III–IV), chronic GVHD (39.07%). Causes of death are detailed, with disease recurrence (13.27%) being the most common, followed by multiple organ failure (6.97%), infection (4.42%), and respiratory failure (2.72%), among others (**Table 2**).

**Table 2:** Post−transplant outcomes of the study cohort.

Factor	Level	Overall
		N=558
aGVHD (n, %)		246 (44.09%)
aGVHD grade (n, %)	I~II	173 (31.00%)
	III~IV	73 (13.08%)
cGVHD (n, %)		218 (39.07%)
CIR [Rate, 95%CI]	3-year	29.7 (24.2~31.7)
NRM [Rate, 95%CI]	3-year	11.1 (7.46~12.4)
Death reasons (n, %)	Disease Recurrence	78 (13.27%)
	Multiple organ failure	41 (6.97%)
	Infection	26 (4.42%)
	Respiratory failure	16 (2.72%)
	Pulmonary Hemorrhage	4 (0.68%)
	Intracerebral Hemorrhage (ICH)	4 (0.68%)
	Heart Failure (HF)	3 (0.51%)
	GVHD	1 (0.17%)
	Hemorrhagic Shock	1 (0.17%)
	Progressive Disease	1 (0.17%)
	Hemorrhagic Stroke	1 (0.17%)
	Ventricular Fibrillation	1 (0.17%)

### Germline predisposition gene mutations in patients with hematologic diseases

**Figure 1:**
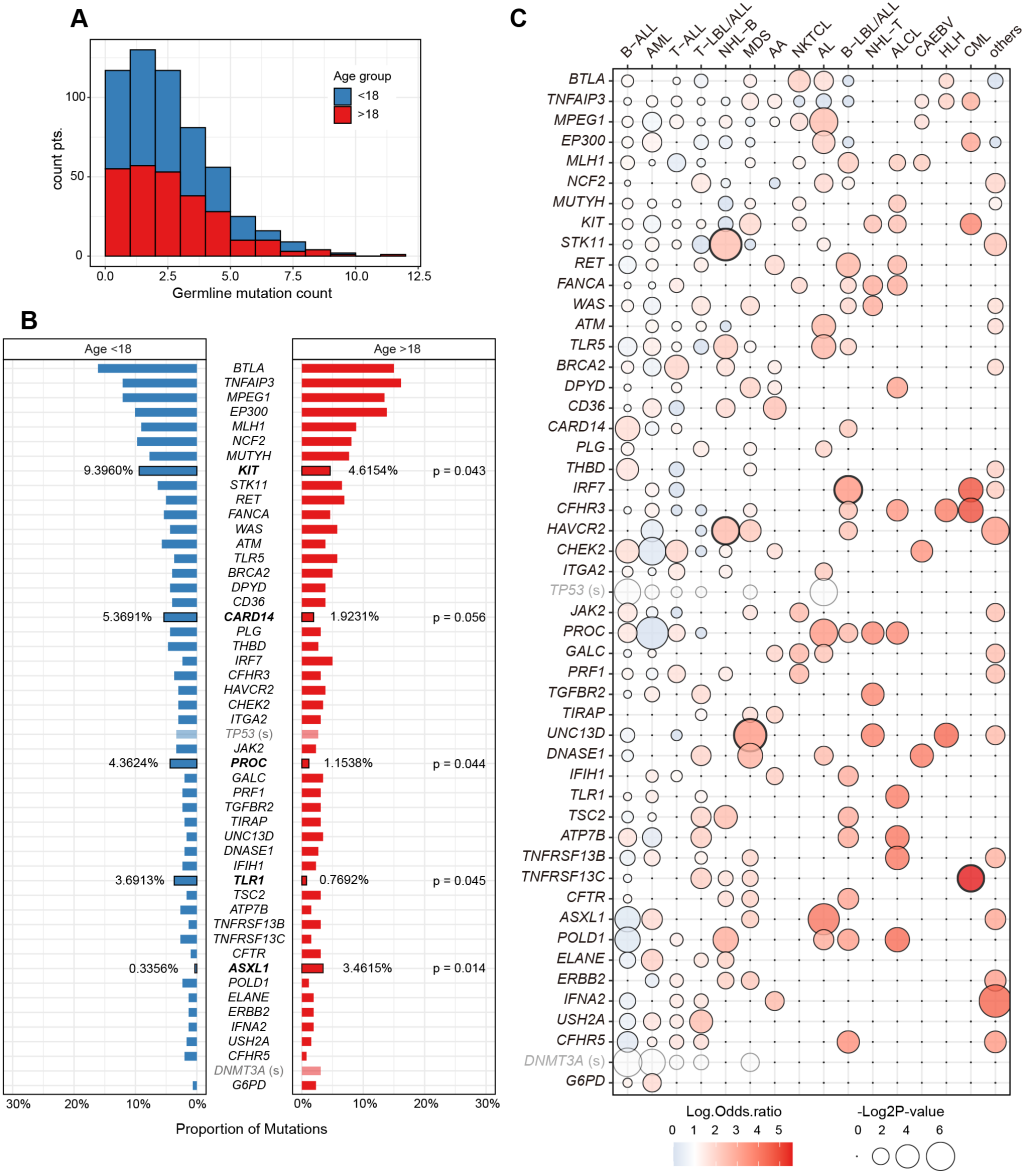
Germline genetic susceptibility mutation landscape in pediatric and adult patients. **(A)** Histograms showing the distribution of the number of germline susceptibility mutation sites carried by pediatric (<18 years) and adult (>=18 years) patients. **(B)** Top 50 germline susceptibility genes with differential distribution between pediatric and adult patients. **(C)** Heatmap depicting the distribution of specific germline susceptibility gene mutations across different hematologic malignancy types. Enrichment of specific genes in certain diseases is highlighted. Mutations in TP53 and DNMT3A were verified as high-VAF somatic mutations and are excluded from further germline mutation analysis.

All 558 patients in the cohort underwent germline predisposition gene testing prior to HSCT. The median number of germline predisposition gene mutation sites carried by each patient was 3 (range: 0–12; IQR: 2–4). No significant difference was observed in the number of germline predisposition gene mutation sites between adult and pediatric patients (p-value = 0.42; **Figure 1A**). The top 10 most frequently detected germline predisposition gene mutations were *BTLA* (15.6%, n = 87), *TNFAIP3* (14.0%, n = 78), *MPEG1* (12.7%, n = 71), *EP300* (11.8%, n = 66), *MLH1* (9.0%, n = 50), *NCF2* (9.0%, n = 50), *MUTYH* (7.7%, n = 43), *KIT* (7.2%, n = 40), *STK11* (6.5%, n = 36), and *RET* (5.9%, n = 33).

Analysis of the top 50 genes by mutation frequency revealed differences in mutation patterns between pediatric and adult patients. Five genes showed differential distribution between the two groups (**Figure 1B**). *KIT*, *CARD14*, *PROC*, and *TLR1* mutations were more frequent in pediatric patients than in adult patients (*KIT*: 28/298 vs 12/260, p = 0.043; *CARD14*: 16/298 vs 5/260, p = 0.056; *PROC*: 13/298 vs 3/260, p = 0.044; *TLR1*: 11/298 vs 2/260, p = 0.045). In contrast, *ASXL1* mutations were more frequent in adult patients than in pediatric patients (9/260 vs 1/298, p = 0.014). No association was observed between these germline predisposition gene mutations and HSCT outcomes (data not shown).

In addition, detected germline inborn errors of immunity (IEI) gene mutations were classified according to the 2022 classification of Human Inborn Errors of Immunity (17). An oncoplot of IEI gene mutations is shown in **Supplementary Figure 1**. A total of 372 patients (66.7%) were found to carry IEI gene mutations (median number of mutated genes = 1, IQR: 0–2; median number of mutation sites = 1, IQR: 0–2). The most frequently identified IEI categories were congenital defects of phagocyte number or function (19.9%, n = 111), autoinflammatory disorders (18.1%, n = 101), and combined immunodeficiencies with associated or syndromic features (15.2%, n = 85). No significant differences were observed between pediatric and adult patients in the distribution of germline IEI gene mutation categories (**Table 2**).

Furthermore, differences in germline predisposition gene mutations among patients with different disease types were examined (**Figure 1C**). Germline *STK11* and *HAVCR2* mutations were significantly enriched in patients with B-NHL (*STK11*: odds ratio = 4.77, p-value = 0.0099; *HAVCR2*: odds ratio = 5.10, p-value = 0.034). Germline *IRF7* mutations were significantly enriched in patients with B-LBL/ALL (odds ratio = 9.74, p-value = 0.030). Germline *UNC13D* mutations were significantly enriched in patients with MDS (odds ratio = 9.51, p-value = 0.0082). In CML, germline *TNFRSF13C* mutations were significantly enriched (odds ratio = 47.48, p-value = 0.043).

Further analyses explored the association between disease-specific germline mutations and HSCT outcomes. The *HAVCR2* mutation enriched in B-NHL was potentially associated with poorer DFS (DFS: B-NHL with *HAVCR2*-mut, p-value = 0.0024; OS: B-NHL with *HAVCR2*-mut, p-value = 0.26; **Supplementary Figure 2A**). Among the three B-NHL patients carrying *HAVCR2* mutations, two received transplants from unrelated donors and one from a related haploidentical donor. All three patients experienced relapse within one year after HSCT, and relapse occurred within three months after transplantation in two patients. Patients P157 and P624 received CD19 CAR-T therapy after relapse; disease remission was achieved in P624, whereas P157 showed a poor response to CAR-T therapy and died due to relapse. Patient P249 received chemotherapy and donor lymphocyte infusion (DLI) after relapse; disease remission was not achieved, and the cause of death was pulmonary hemorrhage. Insufficient data were available to demonstrate a significant impact of donor selection or *HAVCR2* donor genotype on prognosis (**Supplementary Figures 2B, C**).

Similarly, we found that MDS patients carrying *UNC13D* mutations might be associated with poorer outcomes (DFS: MDS with *UNC13D*-mut, p-value = 0.06; OS: MDS with *UNC13D*-mut, p-value = 0.055; **Supplementary Figure 3A**). Among the three MDS patients carrying *UNC13D* mutations, patient P301 received transplantation from a related donor carrying a *UNC13D*-mut allele; no relapse was observed, and the patient died from stroke. Patient P392 also received transplantation from a related donor whose *UNC13D* status was wild type and has not relapsed to date. Patient P739 received transplantation from an unrelated donor and relapsed within three months after HSCT; the patient showed a poor response to chemotherapy after relapse and subsequently died (**Supplementary Figure 3B**). We further evaluated the impact of donor *UNC13D* genotype on outcomes in the entire cohort. Patients who received transplantation from *UNC13D*-wild-type related donors tended to have better outcomes, although no statistically significant differences were observed (DFS: *UNC13D*-mut with wild-type related donor, p-value = 0.32; OS: *UNC13D*-mut with wild-type related donor, p-value = 0.40; **Supplementary Figure 3C**). Specifically, among *UNC13D*-mut carriers, the six patients who received grafts from related donors with wild-type *UNC13D* all remained alive and relapse-free. In contrast, of the six patients who received transplants from unrelated donors, one died from relapse and another died without relapse. Furthermore, the one patient who received a transplant from a related donor carrying an *UNC13D* mutation died without relapse.

We also analyzed the germline status of *TP53* variants and the clinical outcomes of patients carrying mutations in the three other predisposition genes (*RUNX1, GATA2, ETV6*) that were previously highlighted by the 2017 Journal of Clinical Oncology report. To confirm the germline origin of detected *TP53* variants, we performed validation using nail and oral swab samples from all 17 patients initially identified with *TP53* mutations. All 17 variants were confirmed to be somatic in origin. Among six patients carrying *RUNX1* mutations, no significant differences in transplant outcomes were observed (DFS: p-value = 0.27; OS: p-value = 0.47, data not show). One patient each carried *GATA2* and *ETV6* mutations. The patient with the *ETV6* mutation remained alive at the last follow-up, whereas the patient with the *GATA2* mutation relapsed and died.

### Unrelated donor selection improves HSCT outcomes in patients with homozygous germline predisposition gene mutations

**Figure 2:**
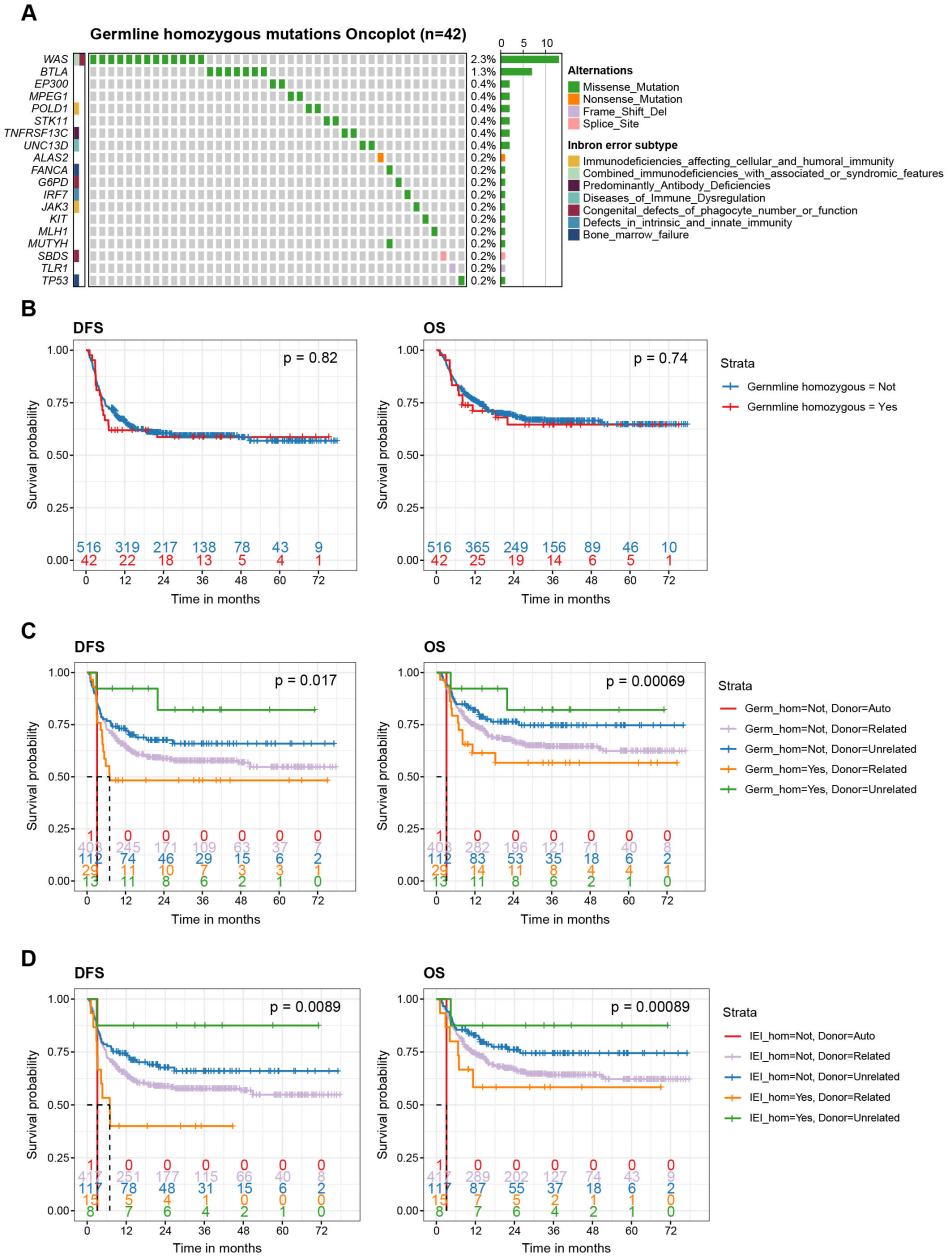
Impact of germline homozygous mutations and donor selection on transplant outcomes. **(A)** Oncoplot showing the recurrent germline homozygous mutation genes identified in the cohort. **(B)** Kaplan-Meier curves comparing Disease-Free Survival (DFS) and Overall Survival (OS) between patients with and without germline homozygous mutations. **(C)** Kaplan-Meier curves for DFS and OS based on donor type (Related vs. Unrelated) and the presence of germline homozygous mutations (Homozygous-mut vs. non-homozygous). **(D)** Kaplan-Meier curves for DFS and OS focusing on patients with homozygous Inborn Errors of Immunity (IEI) mutations, stratified by donor type (Related vs. Unrelated).

In our cohort, 42 patients (7.5%) were identified as carrying homozygous germline predisposition gene mutations. Recurrent homozygous mutations were observed in *WAS* (n = 13), *BTLA* (n = 7), *EP300* (n = 2), *MPEG1* (n = 2), *POLD1* (n = 2), *STK11* (n = 2), *TNFRSF13C* (n = 2), and *UNC13D* (n = 2) (**Figure 2A**). No significant differences in prognosis were observed between patients with and without homozygous germline predisposition mutations (DFS: p-value = 0.82; OS: p-value = 0.74; **Figure 2B**), suggesting that HSCT may improve outcomes in patients carrying homozygous germline predisposition gene mutations.

We further analyzed the impact of donor selection on outcomes in patients with homozygous germline predisposition mutations. Prognosis differed according to donor type among patients with homozygous germline mutations (DFS: p-value = 0.017; OS: p-value = 0.00069; **Figure 2C**). Among patients carrying homozygous germline mutations, selection of unrelated donors significantly improved DFS (DFS: p-value = 0.031; OS: p-value = 0.092). Patients with homozygous germline mutations who received transplantation from related donors had a 3-year DFS of 48.3% (95% CI: 33.1–70.4%) and a 3-year OS of 56.7% (95% CI: 40.7–79.0%), whereas those who received transplantation from unrelated donors had a 3-year DFS of 82.1% (95% CI: 62.1–100%) and a 3-year OS of 82.1% (95% CI: 62.1–100%).

Similar results were observed in patients carrying homozygous IEI mutations. In our cohort, 23 patients carried homozygous IEI mutation sites, and those who received transplantation from unrelated donors had significantly better outcomes than those who received transplantation from related donors (DFS: p = 0.0089; OS: p = 0.00089; **Figure 2D**). Patients carrying homozygous IEI germline mutations, selection of unrelated donors significantly improved DFS (DFS: p-value = 0.047; OS: p-value = 0.18). Among patients with homozygous IEI mutations, the 3-year DFS was 87.5% (95% CI: 67.3–100%) in those receiving unrelated donor transplants, compared with 40.0% (95% CI: 21.5–74.3%) in those receiving related donor transplants. Consistent findings were observed for OS, with a 3-year OS of 87.5% (95% CI: 67.3–100%) in patients receiving unrelated donor transplants, compared with 58.3% (95% CI: 37.4–90.9%) in those receiving related donor transplants.

### Poor post-HSCT outcomes in patients with germline mutations affecting cellular and humoral immunity

**Figure 3:**
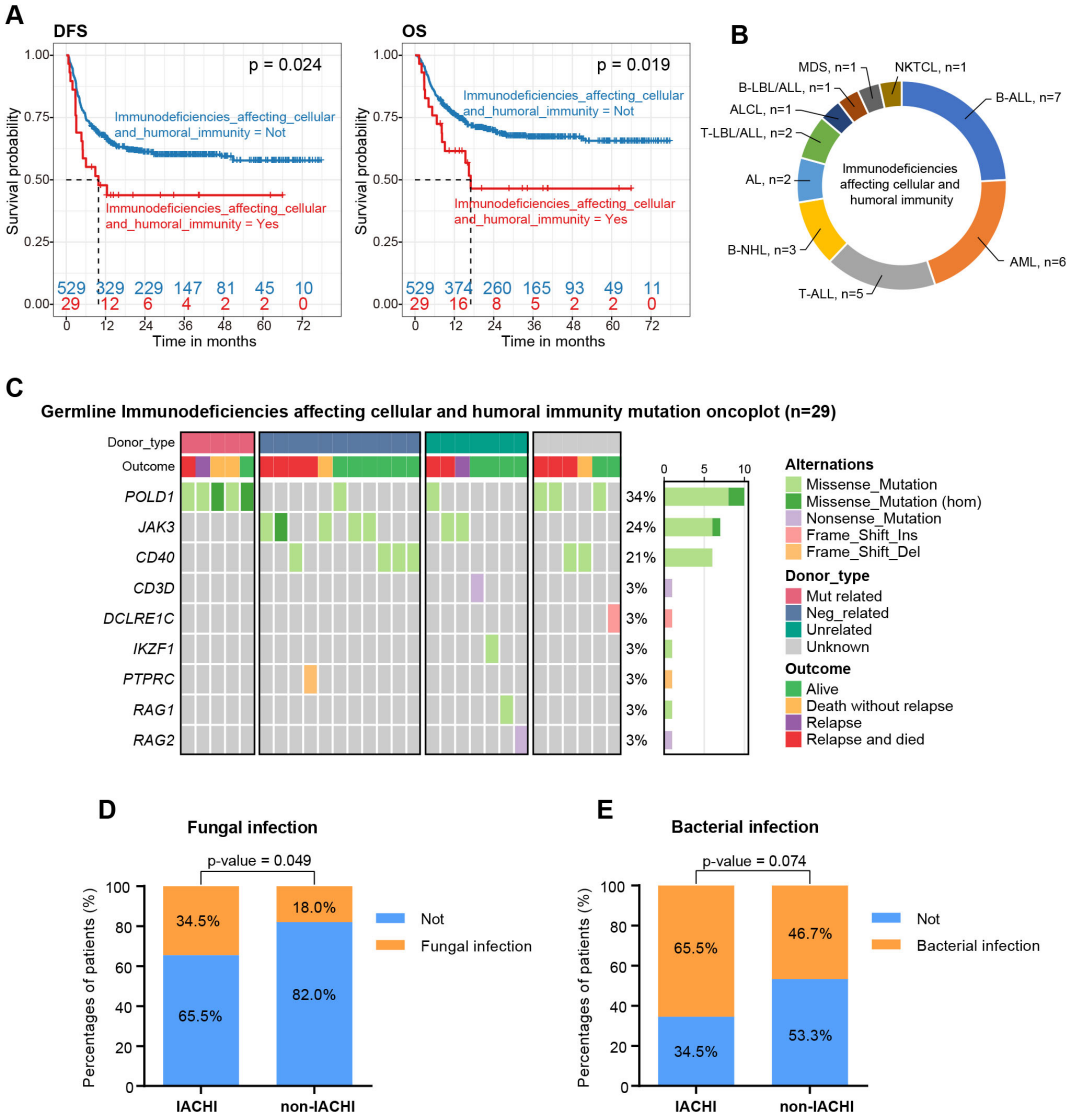
Poor post-transplant outcomes in carriers of germline mutations in Immunodeficiencies Affecting Cellular and Humoral Immunity (IACHI). **(A)** Kaplan-Meier curves showing significantly inferior DFS and OS in patients carrying germline IACHI mutations compared to non-carriers. **(B)** Pie plot illustrating the distribution of various hematologic malignancy types among the 29 patients carrying IACHI germline mutations. **(C)** Oncoplot detailing the specific IACHI gene mutation profile of the 29 carriers and their clinical outcomes post-HSCT, stratified by donor type and donor germline status (Mut_related: related donor with IACHI mutation; Neg_related: related donor without IACHI mutation; Unrelated: unrelated donor). **(D)** Bar charts showing the incidence of fungal infection in patients with germline IACHI mutations compared to non-IACHI patients. **(E)** Bar charts showing the incidence of bacterial infection in IACHI mutated patients compared to non-IACHI patients.

We further analyzed the impact of different categories of IEI gene mutations on patient outcomes. The detailed distribution of IEI mutation categories is presented in **Table 3**. Patients carrying germline mutations classified as immunodeficiencies affecting cellular and humoral immunity (IACHI) had significantly poorer outcomes (DFS: HR = 1.79, 95% CI: 1.07–2.97, p-value = 0.024; OS: HR = 1.90, 95% CI: 1.10–3.28, p-value = 0.019; **Figure 3A**). In this subgroup, the median DFS was 9.83 months (95% CI: 5–NA months), and the median OS was 17.0 months (95% CI: 8.25–NA months).

**Table 3:** Comparison of Inborn Errors of Immunity (IEI) mutation categories between pediatric and adult patients.

Factor	Overall	<18	≥18	p
	N=558	N=298	N=260	
IEI status (%)	372 (66.7%)	197 (66.1%)	175 (67.3%)	0.834
IEI count by site (median [IQR])	1.00[0.00,2.00]	1.00[0.00,2.00]	1.00[0.00,2.00]	0.524
IEI count by group (median [IQR])	1.00[0.00,2.00]	1.00[0.00,2.00]	1.00[0.00,2.00]	0.647
Immunodeficiencies affecting cellular and humoral immunity (%)	29 (5.2%)	16 (5.4%)	13 (5.0%)	0.996
Combined immunodeficiencies with associated or syndromic features (%)	85 (15.2%)	46 (15.4%)	39 (15.0%)	0.98
Predominantly Antibody Deficiencies (%)	5 (0.9%)	2 (0.7%)	3 (1.2%)	0.878
Diseases of Immune Dysregulation (%)	43 (7.7%)	18 (6.0%)	25 (9.6%)	0.155
Congenital defects of phagocyte number or function (%)	111 (19.9%)	56 (18.8%)	55 (21.2%)	0.555
Defects in intrinsic and innate immunity (%)	67 (12.0%)	37 (12.4%)	30 (11.5%)	0.851
Autoinflammatory Disorders (%)	101 (18.1%)	50 (16.8%)	51 (19.6%)	0.448
Complement Deficiencies (%)	58 (10.4%)	36 (12.1%)	22 (8.5%)	0.208
Bone marrow failure (%)	88 (15.8%)	49 (16.4%)	39 (15.0%)	0.726

In our cohort, 29 patients carried germline mutations belonging to this category. These patients were distributed across multiple hematologic disease types, with the main diagnoses including 7 cases of B-ALL, 6 cases of AML, 5 cases of T-ALL, 3 cases of B-NHL, 2 cases of AL, and 2 cases of T-LBL/ALL (**Figure 3B**). The proportion of male patients was significantly higher in this group (IACHI vs non-IACHI: 24 [82.8%] vs 305 [57.7%], p-value = 0.013). In addition, a significantly higher incidence of fungal infections was observed after HSCT (IACHI vs non-IACHI: 10 [34.5%] vs 95 [18.0%], p-value = 0.049; **Figure 3D**). An increased incidence of bacterial infections was also observed, although the difference did not reach statistical significance (IACHI vs non-IACHI: 19 [65.5%] vs 247 [46.7%], p-value = 0.074; **Figure 3E**). Detailed information on specific germline IACHI mutations is shown in **Figure 3C**.

We further found that patients who received transplants from related donors carrying the same IACHI germline mutations (Mut_related) had poor outcomes. Among the five patients who received transplants from Mut_related donors, one patient relapsed and died, one relapsed and is currently alive, and two died without relapse due to infection and multiple organ failure. Patient P322 received chemotherapy and CD7 CAR-T reinfusion after relapse, achieved remission, subsequently relapsed again, and died of heart failure. Patient P428 received chemotherapy and DLI after relapse and is currently alive. Patients P614 and P740 died of infection and multiple organ failure without evidence of relapse.

In contrast, patients who received transplants from related donors without IACHI germline mutations (Neg_related, n = 11) had better outcomes than those in the Mut_related group. Four patients relapsed and died, all due to disease relapse; one patient died without relapse due to infection and graft-versus-host disease (GVHD); and six patients remain alive without relapse. Outcomes in patients who received transplants from unrelated donors were similar to those in the Neg_related group. Among seven such patients, three had poor outcomes: two relapsed and died (one due to disease relapse and one due to respiratory failure), and one relapsed but remains alive. These findings suggest that screening related donors for IACHI germline mutations may improve outcomes in patients carrying IACHI germline mutations.

### Multivariable analysis of factors influencing post−transplant outcomes

To identify independent prognostic factors for DFS and OS, we performed a multivariable Cox proportional hazards regression including the following covariates: age group, conditioning regimen, aGVHD, cGVHD, homozygous IEI mutations (IEI hom), and IACHI. The results are summarized in **Table 4**.

**Table 4:** Multivariable Cox regression analysis of factors associated with DFS and OS.

Factor	Ref	DFS			OS		
		HR	95%CI	p-value	HR	95%CI	p-value
Age group (>18)	<18	1.245	0.9546~1.6237	0.1059	1.467	1.0895~1.9755	0.0116
Conditioning regimen (TBI-based)	BU-based	1.044	0.7986~1.3636	0.7549	1.08	0.8012~1.4564	0.6127
Conditioning regimen (others)	BU-based	1.41E-06	0~Inf	0.9932	1.48E-06	0~Inf	0.9941
aGVHD (Yes)	No	1.148	0.871~1.5118	0.3279	1.321	0.971~1.7984	0.0763
cGVHD (Yes)	No	0.4343	0.3199~0.5895	8.82E-08	0.4015	0.2842~0.5673	2.29E-07
IEI hom (Yes)	No	1.292	0.6787~2.4585	0.4357	1.12	0.5201~2.4111	0.7723
Immunodeficiencies affecting cellular and humoral immunity (Yes)	No	1.617	0.9641~2.7118	0.0686	1.843	1.0583~3.2094	0.0307
		Global p-value = 0.00001			Global p-value = 0.000006		

For DFS, cGVHD remained the only significant independent protective factor (HR = 0.4343, 95% CI: 0.3199–0.5895, p-value = 8.82×10⁻^8^). None of the other covariates reached statistical significance. For OS, cGVHD was again independently associated with improved survival (HR = 0.4015, 95% CI: 0.2842–0.5673, p-value = 2.29×10⁻^7^). In addition, IACHI gene mutations independently predicted worse OS (HR = 1.843, 95% CI: 1.0583–3.2094, p-value = 0.0307). Age >18 years also showed a significant association with inferior OS (HR = 1.467, p-value = 0.0116). The global p-values for the DFS and OS models were 1×10⁻^5^ and 6×10⁻^6^.

These findings suggest that cGVHD is an independent favorable prognostic factor, while deficits in cellular and humoral immunity independently increase the risk of death after transplantation.

## Discussion

In recent years, the role of germline predisposition genes in hematologic malignancies in the prognostic assessment of HSCT has received increasing attention (1, 18–20). Systematic genetic screening of patients prior to transplantation facilitates the identification of high-risk individuals and provides a basis for individualized treatment strategies.

In this study, all 558 patients underwent germline predisposition gene testing before HSCT. The results showed that up to 94.4% of patients (527/558) carried pathogenic and likely pathogenic variants germline predisposition variants. In unselected hematologic malignancy populations, reported rates of pathogenic/likely pathogenic germline variants typically range from 5% to 10% (21, 22), and among allogeneic HSCT recipients, clinically relevant germline variants have been identified in approximately 17.8% of patients (23). The much higher prevalence in our cohort is likely explained by the unique composition of our study population: 59.9% of patients had experienced relapse before the current HSCT, and 24.4% were undergoing a second transplantation. Therefore, our findings reflect the high−risk nature of this real−world transplant cohort and should not be generalized to unselected hematologic malignancy patients. Independent validation in larger, more diverse cohorts is warranted. The ten most frequently detected genes were *BTLA*, *TNFAIP3*, *MPEG1*, *EP300*, *MLH1*, *NCF2*, *MUTYH*, *KIT*, *STK11*, and *RET*. Among these, *BTLA* and *TNFAIP3* variants were particularly prevalent, accounting for 21.3% and 18.7% of all detected variants, respectively. These genes play key roles in immune regulation, DNA repair, and apoptotic pathways (17).

Previous report identified *TP53*, *RUNX1*, *GATA2*, and *ETV6* as being associated with the development of hematologic malignancies (24). Our finding that all detected *TP53* variants were somatic, despite high VAFs, underscores the importance of validation using germline-appropriate tissues in cohorts with leukemia-predisposition genes. Regarding *RUNX1*, *GATA2*, and *ETV6*, the limited number of carriers in our cohort precluded definitive conclusions. Although no significant difference in transplant outcomes was observed for *RUNX1* mutation carriers, the sample size (n=6) was small. Similarly, the contrasting outcomes for the single *GATA2* (death) and *ETV6* (alive) carriers highlight the need for larger, multicenter studies to evaluate the impact of these germline mutations on HSCT outcomes.

In our cohort, patients carried a median of three mutated genetic loci, indicating that germline predisposition gene variants should be considered in both pediatric and adult populations. Significant differences in germline gene enrichment were observed between pediatric and adult patients. Germline mutations in *KIT*, *CARD14*, *PROC*, and *TLR1* were significantly more frequent in pediatric patients, whereas germline *ASXL1* mutations were significantly more prevalent in adult patients. As a key regulator of hematopoietic stem cell proliferation and differentiation, the high frequency of *KIT* mutations in pediatric patients may be closely associated with imbalance of the hematopoietic microenvironment during early developmental stages (25). *CARD14* participates in the regulation of the NF-κB signaling pathway, and mutations occurring during the immature phase of immune system development in children may more readily trigger abnormal inflammatory responses and cell proliferation (26). Mutations in *PROC* may lead to disturbances in the balance between coagulation and fibrinolysis, suggesting a potential bleeding risk in pediatric patients (27). In contrast, the enrichment of *ASXL1* mutations in adults may affect epigenetic regulation and clonal evolution of hematopoietic stem cells (28–30). These findings suggest that the underlying pathogenic mechanisms may differ by age.

In disease-specific analyses, germline *UNC13D* mutations were enriched in MDS, whereas enrichment of *TNFRSF13C* mutations was observed in CML. Germline *IRF7* mutations were enriched in B-ALL/LBL, while enrichment of germline *STK11* and *HAVCR2* mutations was observed in B-cell non-Hodgkin lymphoma. The enrichment of distinct germline mutations across different hematologic malignancies further indicates that the genomic characteristics vary among disease subtypes. Germline *UNC13D* mutations are generally considered to be associated with HLH (31). However, the enrichment of *UNC13D* mutations observed in patients with MDS may be related to defects in cytotoxic T lymphocyte- and natural killer (NK) cell-mediated immune responses (32). Such defects may impair immune surveillance, thereby facilitating immune escape of malignant MDS cells. The enrichment of *TNFRSF13C* germline mutations in CML may be associated with compromised immune surveillance through impaired B-cell maturation, survival, or function (33). These findings provide a theoretical basis for future precision diagnosis and treatment selection based on molecular biomarkers. Notably, the enrichment of *STK11* mutations in B-cell non-Hodgkin lymphoma suggests that this gene may influence tumor initiation and progression through regulation of the cell cycle and DNA repair pathways (34–36). This observation expands the research scope of *STK11* in hematologic malignancies and contrasts with its tumor-suppressive role in solid tumors, suggesting potential functional heterogeneity across different tumor types. In addition, the enrichment of *HAVCR2*, an immune checkpoint molecule, in lymphoma further supports the importance of immune escape mechanisms in disease progression (37). Although not all of these mutations have prognostic value, their association with poorer outcomes in specific diseases (**Supplementary Figures 2**, **3**) highlights the potential utility of genotypic classification in risk stratification.

In our cohort, 42 patients (7.5%) carried homozygous germline predisposition gene mutations, among whom 23 patients harbored homozygous IEI mutations. The eight most frequently recurrent genes were *WAS*, *BTLA*, *EP300*, *MPEG1*, *POLD1*, *STK11*, *TNFRSF13C*, and *UNC13D*. Previous case series have reported that selecting related donors carrying identical germline mutations markedly increases the risk of transplant failure and donor-derived leukemia (38).

We did not observe a significant difference in prognosis between patients carrying homozygous germline mutations, which may reflect the ability of HSCT to overcome certain genetic defects. However, further subgroup analyses demonstrated that, compared with related donors, the use of unrelated donors significantly improved DFS in patients harboring homozygous germline mutations (**Figures 2C, D**), consistent with findings reported in the literature (38). Although the sample size in our study was limited, these results nonetheless highlight the importance of donor selection in patients with homozygous germline predisposition gene mutations. For this patient population, incorporating germline predisposition gene mutation screening into donor selection may be considered in the future to facilitate more precise therapeutic decision-making. This observation also provides a new perspective on the clinical significance of IEI gene mutations in HSCT, which warrants validation in larger clinical cohorts.

In this cohort, 372 patients carried IEI-class gene mutations (17), with a median of one mutation per patient (IQR: 0–2). The number of IEI germline mutation categories was not significantly associated with patient prognosis. However, patients harboring mutations in genes related to IACHI exhibited extremely poor survival outcomes, accompanied by a markedly increased risk of infection and relapse (**Figures 3A, D**). This observation is consistent with the core biological functions of this gene category, which are essential for maintaining normal development and function of the adaptive immune system (17). More specifically, an effect of donor genotype was observed. The poorest outcomes occurred in patients receiving grafts from related donors who also carried IACHI mutations (Mut_related), whereas outcomes were improved in those receiving grafts from related donors without such mutations (Neg_related) or from unrelated donors. Although no statistically significant differences were detected in survival analyses due to the limited sample size (data not shown), these findings strongly suggest the importance of targeted germline predisposition gene screening in potential related donors for this high-risk patient group. This approach may represent a simple and effective strategy to improve outcomes in patients carrying germline IACHI gene mutations.

Several limitations of this study should be acknowledged. First, this was a single-center retrospective study and was therefore subject to inherent selection bias. Second, germline genetic testing was based on targeted sequencing, and thus may not capture all variants at the whole-genome level. Finally, the sample sizes of certain subgroups, such as patients harboring specific combinations of mutations, were small, limiting statistical power; therefore, these conclusions require confirmation in larger prospective cohorts. Additionally, given the large number of genes tested and the exploratory nature of the analysis, we did not correct for multiple testing; therefore, some of the observed associations may be chance findings and should be interpreted cautiously. We anticipate conducting prospective, multicenter studies with larger, disease−specific cohorts in the future to validate the role of germline genetic factors in predicting transplant outcomes and optimizing donor selection.

In conclusion, our study demonstrates that germline predisposition genes represent an important determinant of HSCT outcomes. For patients harboring high-risk mutations, such as homozygous IEI-related variants or IACHI-associated mutations, appropriate donor selection may contribute to improved prognosis. These findings underscore the potential value of germline predisposition gene mutations in guiding personalized donor selection for patients undergoing HSCT.

## Data Availability

The raw data supporting the conclusions of this article have been uploaded as **Supplementary Material** (Excel file) via the journal’s submission system. Anonymized data will be shared upon reasonable request to the corresponding author.

## Supplementary material

Supplementary Figure 1 Oncoplot of germline inborn errors of immunity (iei) gene mutations in the cohort.The oncoplot categorized according to the 2022 International Union of Immunological Societies (IUIS) classification. The major IEI categories are annotated.

Supplementary Figure 2 Impact of *HAVCR2* mutation on outcomes in B-NHL patients. **(A)**. Kaplan-Meier curves comparing DFS and OS between B-NHL patients carrying the *HAVCR2* mutation (B-NHL & *HAVCR2*-mut) and other patient groups. **(B)**. Kaplan-Meier curves comparing DFS and OS among B-NHL patients carrying the *HAVCR2* mutation, stratified by *HAVCR2* donor type. **(C)**. Kaplan-Meier curves showing the impact of *HAVCR2* donor type on DFS and OS in carriers.

Supplementary Figure 3 Impact of *UNC13D* mutation on outcomes in MDS patients. **(A)**. Kaplan-Meier curves comparing DFS and OS between MDS patients carrying the *UNC13D* mutation (MDS & *UNC13D*-mut) and other patient groups. MDS patients with *UNC13D* mutation had poorer outcomes. **(B)**. Kaplan-Meier curves comparing DFS and OS among MDS patients carrying the *UNC13D* mutation, stratified by *UNC13D* donor type. **(C)**. Kaplan-Meier curves exploring the impact of *UNC13D* donor type on DFS and OS in carriers.
