# Supplementary material for: Impact of germline predisposition genes to hematologic malignancies on transplant outcomes and donor selection
Source: Front Immunol. 2026 Jul 28;17:1880348. doi: 10.3389/fimmu.2026.1880348 (PMC13457106; doi:10.3389/fimmu.2026.1880348)
Supplement: Supplementary file 1 [file Image1.pdf]

Figure S1

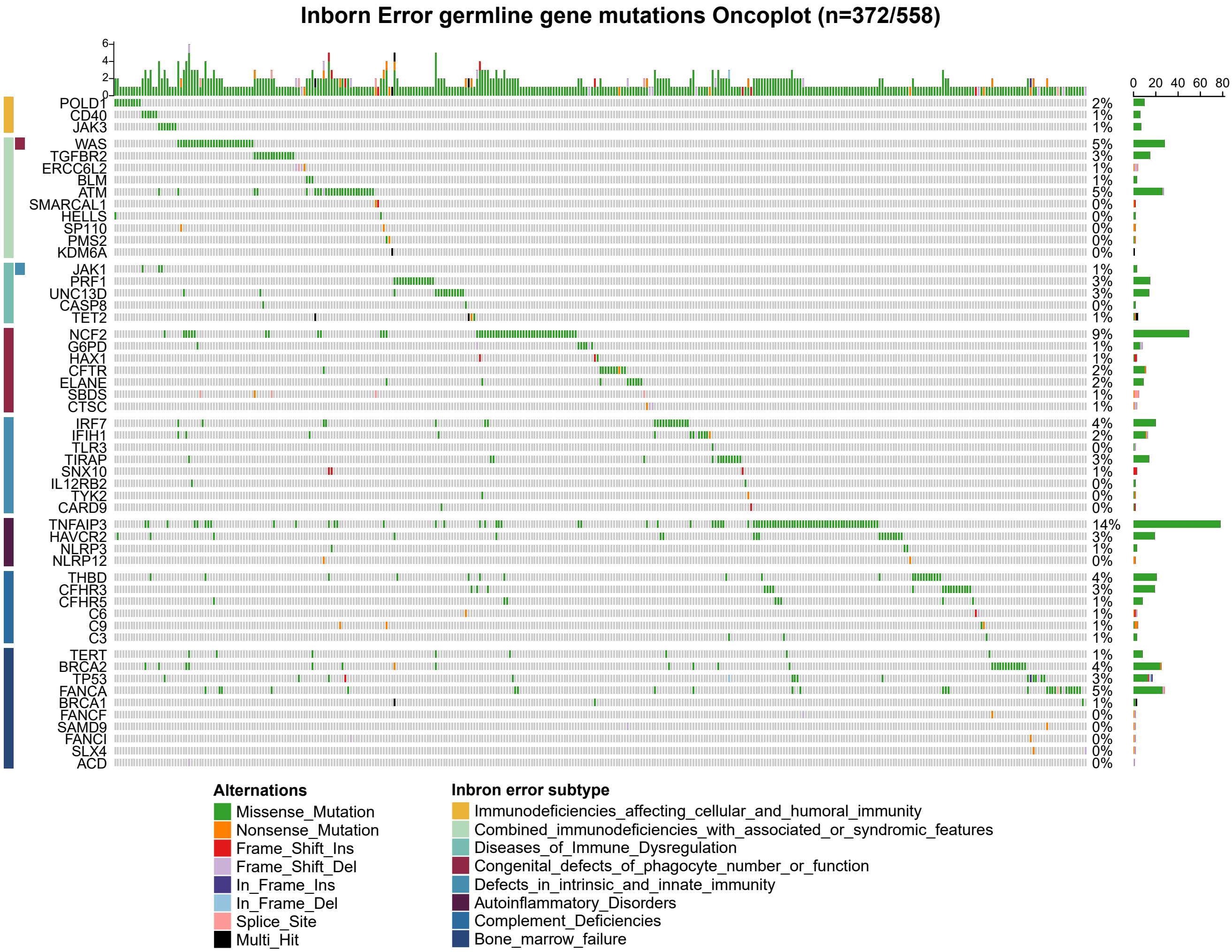

Inborn Error germline gene mutations Oncoplot (n=372/558)
POLD1 2%
CD40 1%
JAK3 1%
WAS 5%
TGFBR2 3%
ERCC6L2 1%
BLM 1%
ATM 5%
SMARCAL1 0%
HELLS 0%
SP110 0%
PMS2 0%
KDM6A 0%
JAK1 1%
PRF1 3%
UNC13D 3%
CASP8 0%
TET2 1%
NCF2 9%
G6PD 1%
HAX1 1%
CFTR 2%
ELANE 2%
SBDS 1%
CTSC 1%
IRF7 4%
IFIH1 2%
TLR3 0%
TIRAP 3%
SNX10 1%
IL12RB2 0%
TYK2 0%
CARD9 0%
TNFAIP3 14%
HAVCR2 3%
NLRP3 1%
NLRP12 0%
THBD 4%
CFHR3 3%
CFHR5 1%
C6 1%
C9 1%
C3 1%
TERT 1%
BRCA2 4%
TP53 3%
FANCA 5%
BRCA1 1%
FANCF 0%
SAMD9 0%
FANCI 0%
SLX4 0%
ACD 0%
0 20 40 60 80
Alternations
Missense_Mutation
Nonsense_Mutation
Frame_Shift_Ins
Frame_Shift_Del
In_Frame_Ins
In_Frame_Del
Splice_Site
Multi_Hit
Inbron error subtype
Immunodeficiencies_affecting_cellular_and_humoral_immunity
Combined_immunodeficiencies_with_associated_or_syndromic_features
Diseases_of_Immune_Dysregulation
Congenital_defects_of_phagocyte_number_or_function
Defects_in_intrinsic_and_innate_immunity
Autoinflammatory_Disorders
Complement_Deficiencies
Bone_marrow_failure
