# Supplementary material for: Impact of germline predisposition genes to hematologic malignancies on transplant outcomes and donor selection
Source: Front Immunol. 2026 Jul 28;17:1880348. doi: 10.3389/fimmu.2026.1880348 (PMC13457106; doi:10.3389/fimmu.2026.1880348)
Supplement: Supplementary file 2 [file Image2.pdf]

Figure S2

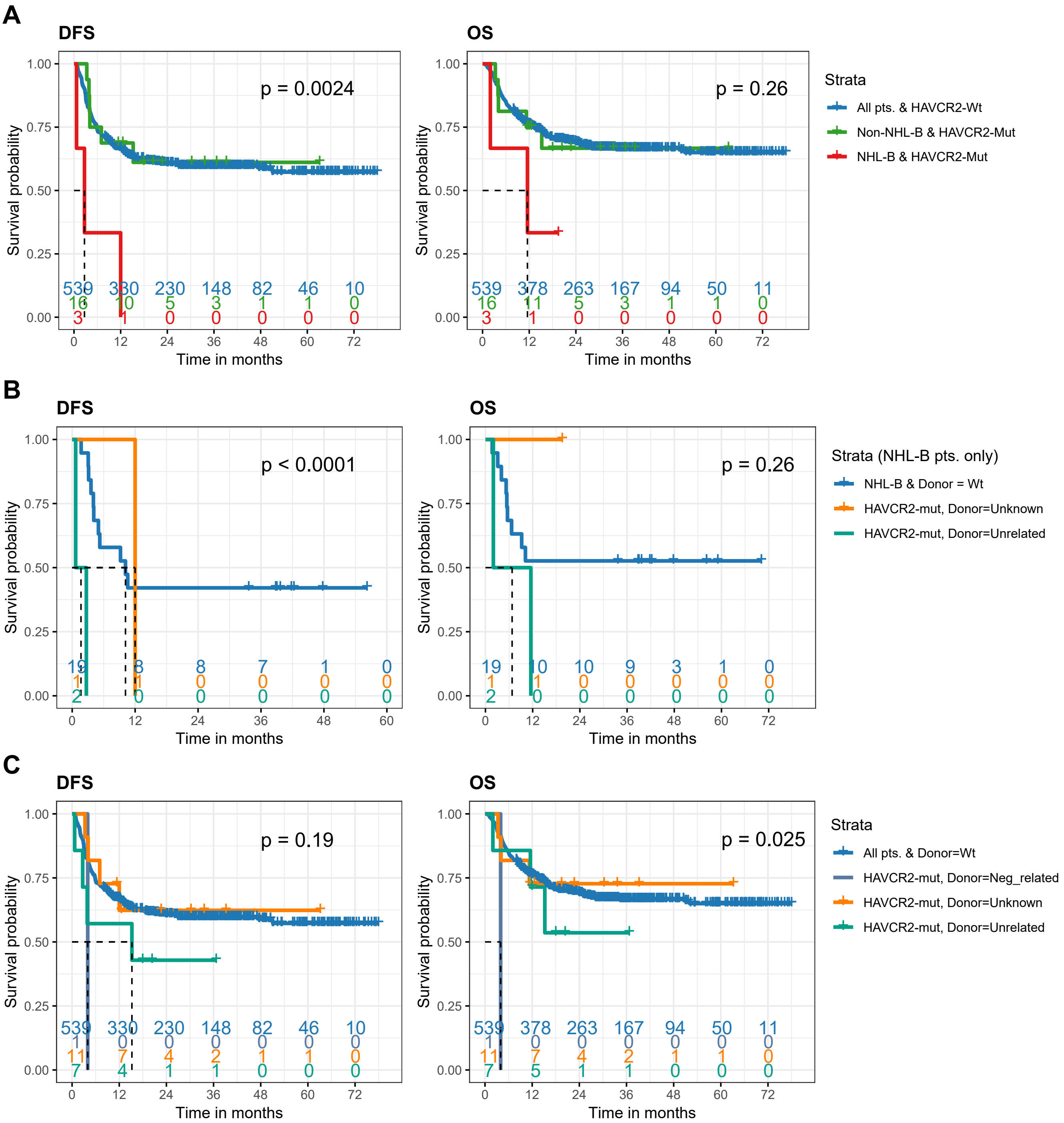
A
DFS
p = 0.0024
OS
p = 0.26
Survival probability
Time in months
Strata
All pts. & HAVCR2-Wt
Non-NHL-B & HAVCR2-Mut
NHL-B & HAVCR2-Mut
B
DFS
p < 0.0001
OS
p = 0.26
Survival probability
Time in months
Strata (NHL-B pts. only)
NHL-B & Donor = Wt
HAVCR2-mut, Donor=Unknown
HAVCR2-mut, Donor=Unrelated
C
DFS
p = 0.19
OS
p = 0.025
Survival probability
Time in months
Strata
All pts. & Donor=Wt
HAVCR2-mut, Donor=Neg_related
HAVCR2-mut, Donor=Unknown
HAVCR2-mut, Donor=Unrelated
