# Supplementary material for: Impact of germline predisposition genes to hematologic malignancies on transplant outcomes and donor selection
Source: Front Immunol. 2026 Jul 28;17:1880348. doi: 10.3389/fimmu.2026.1880348 (PMC13457106; doi:10.3389/fimmu.2026.1880348)
Supplement: Supplementary file 3 [file Image3.pdf]

Figure S3

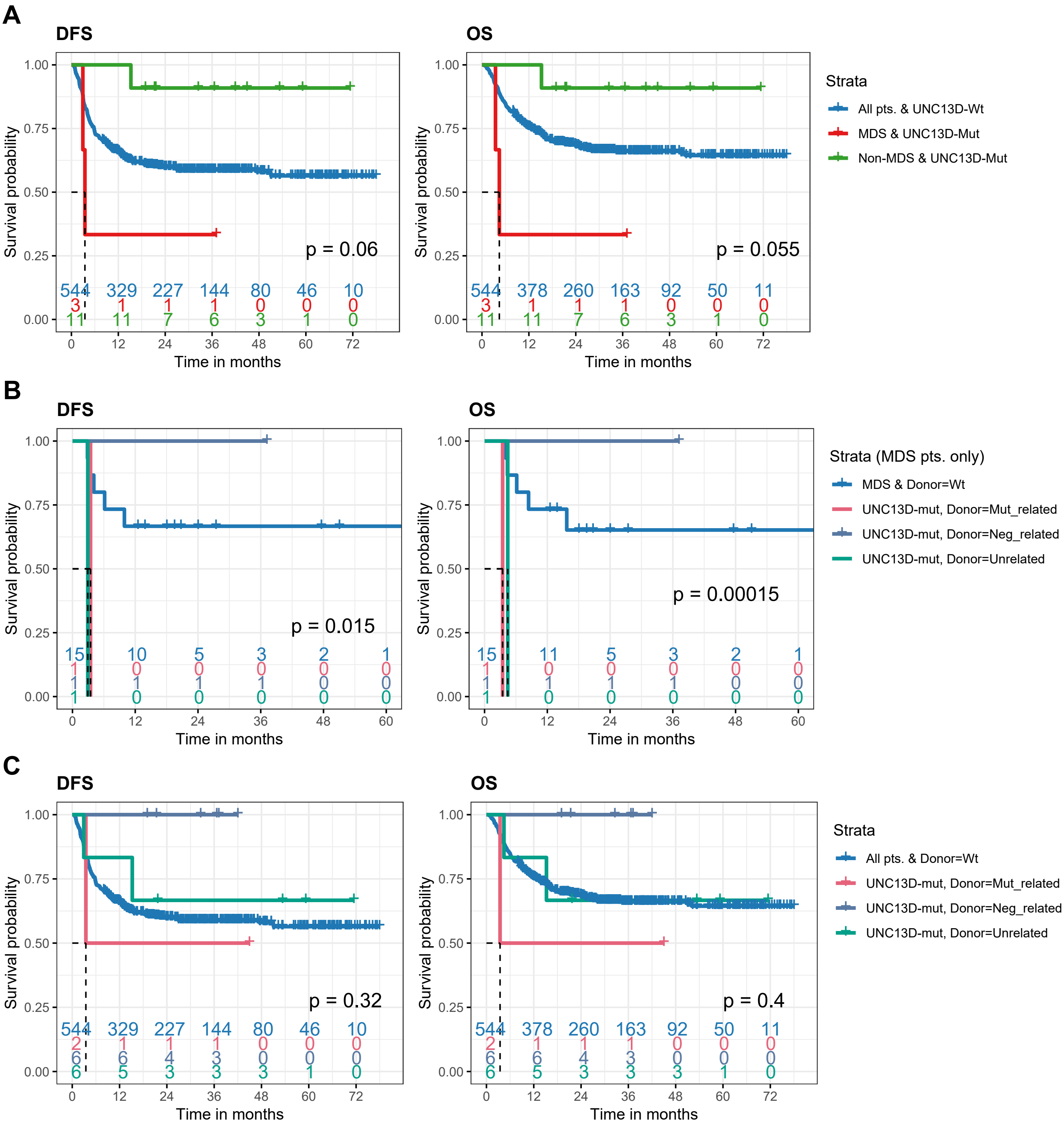

A
DFS
OS
Survival probability
Time in months
p = 0.06
p = 0.055
Strata
All pts. & UNC13D-Wt
MDS & UNC13D-Mut
Non-MDS & UNC13D-Mut
544 329 227 144 80 46 10
3 1 1 1 0 0 0
11 11 7 6 3 1 0
544 378 260 163 92 50 11
3 1 1 1 0 0 0
11 11 7 6 3 1 0
B
DFS
OS
p = 0.015
p = 0.00015
Strata (MDS pts. only)
MDS & Donor=Wt
UNC13D-mut, Donor=Mut_related
UNC13D-mut, Donor=Neg_related
UNC13D-mut, Donor=Unrelated
15 10 5 3 2 1
1 0 0 0 0 0
1 1 1 1 0 0
1 0 0 0 0 0
15 11 5 3 2 1
1 0 0 0 0 0
1 1 1 1 0 0
1 0 0 0 0 0
C
DFS
OS
p = 0.32
p = 0.4
Strata
All pts. & Donor=Wt
UNC13D-mut, Donor=Mut_related
UNC13D-mut, Donor=Neg_related
UNC13D-mut, Donor=Unrelated
544 329 227 144 80 46 10
2 1 1 1 0 0 0
6 6 4 3 0 0 0
6 5 3 3 3 1 0
544 378 260 163 92 50 11
2 1 1 1 0 0 0
6 6 4 3 0 0 0
6 5 3 3 3 1 0
